# Detrimental Role of Nerve Injury-Induced Protein 1 in Myeloid Cells under Intestinal Inflammatory Conditions

**DOI:** 10.3390/ijms21020614

**Published:** 2020-01-17

**Authors:** Hyun Jin Jung, Ju-Hee Kang, Seongwon Pak, Keunwook Lee, Je Kyung Seong, Seung Hyun Oh

**Affiliations:** 1 Interdisciplinary Program in Cancer Biology, College of Medicine, Seoul National University, Seoul 03080, Korea; walkingjin86@gmail.com; 2 Korea Mouse Phenotyping Center, College of Veterinary Medicine, Seoul National University, Seoul 08826, Korea; 3 College of Pharmacy, Gachon University, Incheon 21936, Korea; 4 Department of Biomedical Science, Hallym University, Chuncheon 24252, Korea; 5 Laboratory of Developmental Biology and Genomics, Research Institute of Veterinary Science, BK21 Plus Program for Veterinary Science, Seoul National University, Seoul 08826, Korea

**Keywords:** Ninjurin1, myeloid cells, IL1β, IBD, inflammation

## Abstract

Nerve injury-induced protein 1 (Ninjurin1, Ninj1) is a cell-surface adhesion molecule that regulates cell migration and attachment. This study demonstrates the increase in Ninj1 protein expression during development of intestinal inflammation. Ninj1-deficient mice exhibited significantly attenuated bodyweight loss, shortening of colon length, intestinal inflammation, and lesser pathological lesions than wild-type mice. Although more severe inflammation and serious lesions are observed in wild-type mice than Ninj1-deficient mice, there were no changes in the numbers of infiltrating macrophages in the inflamed tissues obtained from WT and Ninj1-deficient mice. Ninj1 expression results in activation of macrophages, and these activated macrophages secrete more cytokines and chemokines than Ninj1-deficient macrophages. Moreover, mice with conditional deletion of Ninj1 in myeloid cells (Ninj1^fl/fl^; Lyz-Cre+) alleviated experimental colitis compared with wild-type mice. In summary, we propose that the Ninj1 in myeloid cells play a pivotal function in intestinal inflammatory conditions.

## 1. Introduction

Nerve injury-induced protein 1 (Ninjurin1, Ninj1) is identified after a nerve injury, and is considered to be involved in inflammatory responses [1]. In the nervous system, Ninj1 functions have mainly been observed during inflammation, and Ninj1 expression is induced under inflammatory conditions of the central nervous system. Compared to the wild-type (WT) mice, Ninj1 knock-out (KO) mice are less susceptible to experimental autoimmune encephalomyelitis (EAE) [2]. Especially, myeloid cells preferentially express Ninj1, which leads to the modulation of immune cell migration during EAE development [3]. Recent studies reveal that besides the nervous system, Ninj1 regulates several inflammatory diseases. Ninj1 inhibition reduces susceptibility to systemic inflammation, liver damage, and pulmonary inflammation in septic mice [4]. In addition, we have previously reported that Ninj1-deficient mice display a mild lung pulmonary fibrosis phenotype associated with the interaction between macrophages and alveolar epithelial cells [5]. Macrophage infiltration is shown to be crucial for regulating the progression of lung fibrosis [6,7]. However, in the study of lung fibrosis and Ninj1, we demonstrated similar numbers of infiltrating macrophages in bronchoalveolar lavage fluids of bleomycin-treated WT and Ninj1 KO mice for lung fibrosis, indicating that macrophage infiltration does not determine the phenotypic difference between bleomycin-treated WT and Ninj1 KO mice [5].

Dysregulated homeostasis of immune cells is associated with intestinal inflammation [8]. Of the several immune cells that regulate inflammatory responses, macrophages are important during the development of intestinal inflammation [9,10,11]. Inflammatory bowel diseases (IBD), including Crohn’s disease (CD) and ulcerative colitis (UC), are inflammatory disorders that affect the gastrointestinal tract. IBD is a global problem that has emerged in recent years. Development of IBD is associated with environmental and genetic factors as well as immune responses. A recent study reports that IBD involves the differential expression of genes that regulate inflammation and tissue remodeling [12]. Based on the therapeutic effect of immunosuppressive drugs, dysregulation of the immune system has been implicated in IBD [13]. Although previous studies have reported several factors involved in IBD, the epidemiology is still incompletely described.

The production of cytokines and chemokines by monocytes and macrophages at the lesion site is important for the development of inflammation [14]. Several articles demonstrate that IL1β is essential for regulating IBD, and elevated amounts of IL1β have been detected in IBD patients as compared to healthy subjects [15,16]. An animal experiment study reported that treatment with IL1β antagonist significantly diminishes intestinal inflammation [17]. Moreover, IL1β is a master regulator of the inflammatory response, and secretion of IL1β results in upregulation of other proinflammatory cytokines and chemokines such as IL6, TNFα, and CCL2 [18,19]. Therefore, IL1β secretion by activated macrophages is an important factor that drives the intestinal inflammation.

Toll-like receptors (TLRs), NF-κB, p38, and Protein Kinase C (PKC) are reported to be activated for inducing the inflammatory signaling cascade during intestinal inflammation [20]. After triggering of this complex cascade, macrophages produce pro-inflammatory cytokines and chemokines to amplify the inflammatory signal and recruit leukocytes to the lesion site. Ninj1 is reported to modulate the TLR4 signaling cascade, resulting in increased pathogenesis in septic mice [4]. Abundant studies have demonstrated that lipopolysaccharides (LPS) activate TLR families and PKC isoforms [21,22,23,24,25]. Based on their activation requirements, PKC isoforms are categorized into three groups: conventional (α, βI, βII, γ), atypical (ζ, λ/ι), and novel (δ, ε, η, θ) isoforms. PKC activation under inflammatory conditions increases the production of cytokines and chemokines in immune cells. Among the several isoforms, PKCδ/θ contributes to the secretion of cytokines and chemokines in animal models, and is therefore related to inflammatory diseases [26,27]. In the cecal ligation and puncture rat model for sepsis, TLR signaling through PKCδ activation increases the sepsis-induced lung injury, which is evidenced by detecting levels of chemokines in the lungs [28]. PKCδ KO mice exhibit reduced production of cytokines in a lung injury mouse model [29]. Moreover, PKCθ inhibition provides protection to mice from experimental colitis [30].

In this study, we investigated the role of Ninj1 in the pathogenesis of intestinal inflammation by examining Ninj1-deficient mice. We observed that loss of Ninj1 alleviates experimental colitis. Furthermore, mice harboring Ninj1-deficient myeloid cells also exhibit decreased levels of inflammation against colitis.

## 2. Results

### 2.1. Ninj1 Expression Increases under Intestinal Inflammatory Conditions

**Figure 1:**
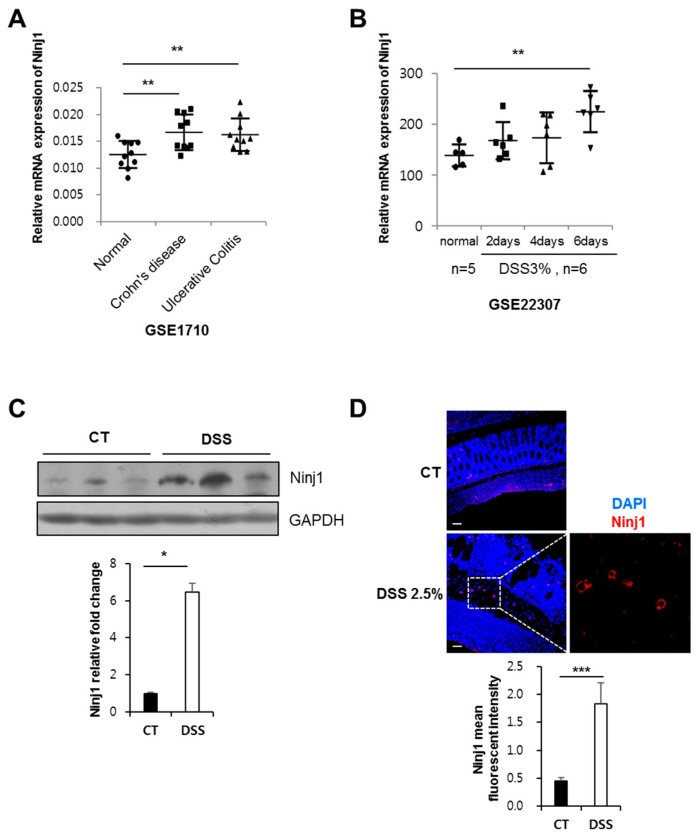
Nerve injury-induced protein 1 (Ninj1) expression in colon during experimental colitis. (**A**) Analysis of Ninj1 expression in colon tissues from ulcerative colitis (UC) and Crohn’s disease (CD) patients. The gene expression data (GSE1710) were obtained from the GEO database. ** *p* < 0.01, Welch’s T test. (**B**) Analysis of Ninj1 expression in colon tissues from mice. The gene expression data (GSE22307) were obtained from the GEO database. ** *p* < 0.01, Welch’s T test. (**C**) Immunoblot analysis of Ninj1 and GAPDH performed on colonic tissues extracted from control (CT) and 1.5% DSS-treated mice; *n* = 3 per group. Similar results were observed in three independent experiments. Densitometry represents relative protein levels of Ninj1. Values are mean ± SD. * *p* < 0.05, Student’s *t*-test. (**D**) Immunofluorescence staining of Ninj1 in colon tissue sections harvested from mice. Representative images are shown. Scale bars, 50 μm. Boxed area is magnified on right. The quantification of Ninj1 immunofluorescence is presented as the mean fluorescence intensity; *n* = 3 per group. Values are mean ± SD. *** *p* < 0.005, Student’s *t*-test.

Since Ninj1 is reported to play a crucial role in immune responses, we analyzed the expression of Ninj1 genes in an IBD patient cohort and in a mouse model of colitis. By analyzing the publicly published mRNA expression profiles, we determined that Ninj1 mRNA expression was greater in colon tissues from patients with CD (*n* = 10) and UC (*n* = 10), as compared to normal controls (*n* = 11) (Figure 1A) [31]. Moreover, Ninj1 mRNA expression was upregulated in the colon of dextran sodium sulfate (DSS)-treated mice, when compared to untreated (normal) mice (Figure 1B) [32]. We further validated Ninj1 expression in mice colon tissues by Western blot analysis. As expected, Ninj1 in colons of DSS-treated mice show increased expression levels as compared to untreated mice (Figure 1C). Immunofluorescence staining revealed that the number of Ninj1^+^ cells significantly increased in colon tissues of DSS-treated mice (Figure 1D). Interestingly, Ninj1 expression in inflamed colon was limited to non-epithelial cells, and cells expressing Ninj1 were mainly localized in the submucosa (Figure 1D). These findings suggest that during colitis, there is increased Ninj1 expression in cells at the sites of inflammation.

### 2.2. Ninj1 Deficiency Alleviates Experimental Colitis

**Figure 2:**
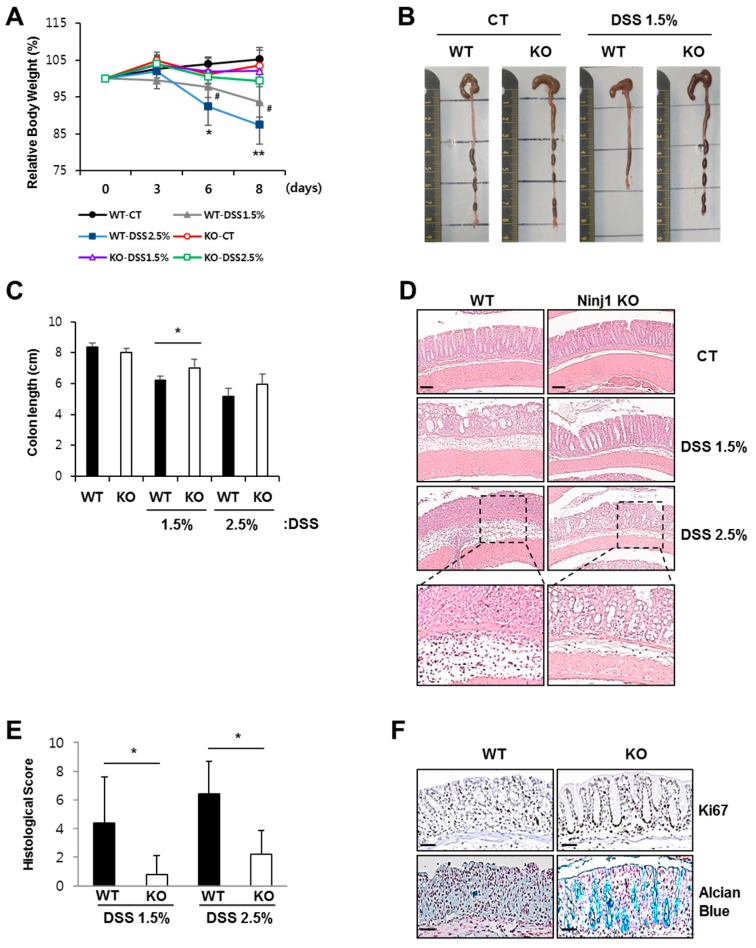
Ninj1 deficiency alleviates the development of intestinal inflammation. (**A**) WT and Ninj1 knock-out (KO) mice were administered 1.5% or 2.5% DSS for 8 days. Bodyweight loss is presented as a percentage of the initial weight ± SD; *n* = 5 per group. Student’s *t*-test comparing 1.5% DSS treated WT mice and 1.5% DSS treated Ninj1 KO mice, ^#^ *p* < 0.05, and comparing 2.5% DSS treated WT mice and 2.5% DSS treated Ninj1 KO mice, * *p* < 0.05, ** *p* < 0.01. Similar results were observed in four independent experiments. (**B**) Mice were sacrificed on day 8 after initiation of DSS treatment. Representative images of colons from WT and Ninj1 KO mice are presented. (**C**) Colon lengths were quantified (mean ± SD). * *p* < 0.05, Student’s *t*-test. (**D**) H&E staining of colon sections was performed, and representative images are presented. Scale bars, 50 μm. Boxed areas are magnified below. (**E**) Submucosal edema, surface epithelial erosion, and crypt damage were evaluated and quantified by histology scores (0–9). Data are presented as mean ± SD. * *p* < 0.05, Student’s *t*-test. (**F**) Ki67 staining of colon sections from 1.5% DSS-treated WT and Ninj1 KO mice were visualized for crypt regeneration. Scale bars, 50 μm (upper panel). Alcian blue staining of colon sections from 1.5% DSS-treated WT and Ninj1 KO mice indicate the epithelial integrity. Scale bars, 50 μm (lower panel). CT, control.

To assess whether Ninj1 has a functional role in colitis, WT and Ninj1 KO mice were administered 1.5% or 2.5% DSS for 8 days, followed by determining the colitis incidences. Ninj1 KO mice exhibit lesser bodyweight loss (Figure 2A) and a considerably longer colon length (Figure 2B,C) as compared to WT mice. Hematoxylin and eosin staining (H&E) analysis reveal less epithelial erosion, crypt destruction, and submucosal edema in the colon of DSS-treated Ninj1 KO mice than DSS-treated WT mice (Figure 2D). The tissue phenotypes associated with colitis were also quantified, and results reveal reduced colitis incidence in DSS-treated Ninj1 KO mice (Figure 2E). Untreated WT and Ninj1 KO mice did not display significant differences in tissue histology features (Figure 2D). Ki-67 and Alcian blue staining were performed to confirm the severity of colitis incidence in Ninj1 KO mice. DSS-treated Ninj1 KO mice had relatively intact epithelia and a greater number of proliferating cells and mucin-containing goblet cells, as compared to DSS-treated WT mice (Figure 2F). Taken together, these results indicate that Ninj1 aggravates experimental colitis.

### 2.3. Macrophages during Colitis Development Show Increased Expression of Ninj1

**Figure 3:**
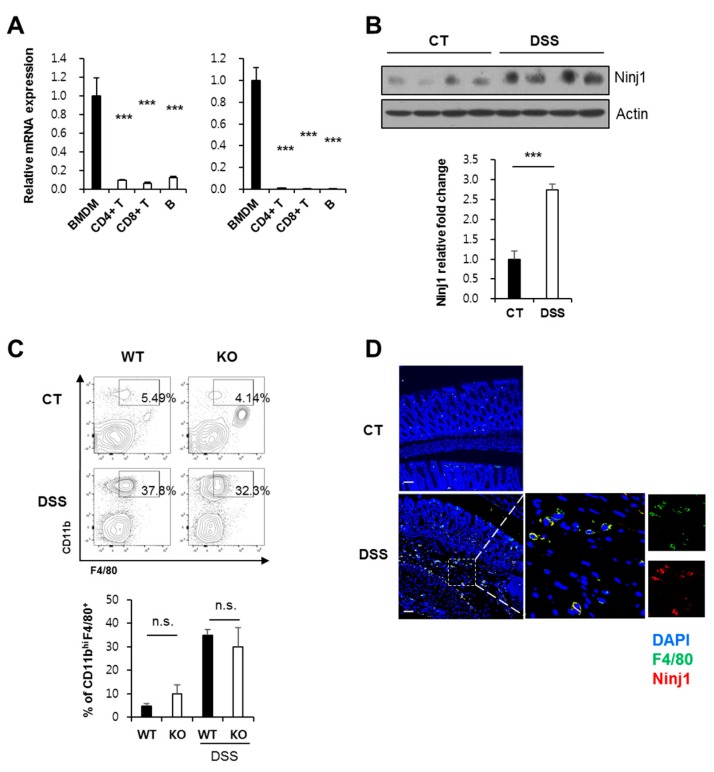
Ninj1 expression in macrophages is enhanced during development of colitis. (**A**) mRNA expression of Ninj1 in the indicated leukocyte populations was analyzed by qRT-PCR. Bone marrow-derived macrophages (BMDMs) were cultivated from bone marrow cells, and CD4^+^ and CD8^+^ T cells and B cells were isolated from spleen using MACS (left panel). Ninj1 expression was determined using lipopolysaccharide (LPS)-treated BMDMs, anti-CD3/anti-CD28-activated CD4^+^ and CD8^+^ T cells, and LPS/BAFF-activated B cells (right panel); *n* = 3 per group. Data represent mean ± SEM. *** *p* < 0.005 compared to the BMDM group, Student’s *t*-test. (**B**) Ninj1 and actin protein expressions were detected in peritoneal macrophages extracted from untreated and 2.5% dextran sodium sulfate (DSS)-treated WT mice; *n* = 4 per group. Densitometry represents relative protein levels of Ninj1. Similar results were observed in three independent experiments. Values expressed are mean ± SD. *** *p* < 0.005, Student’s *t*-test. (**C**) Infiltrating macrophages of DSS-treated mice were analyzed by flow cytometry. Single cell suspensions were isolated from the lamina propria of 1.5% DSS-treated mice or control mice, and stained with surface markers. The representative FACS profiles are shown within viable leukocyte gate (7-AAD^−^ CD45^+^) with frequencies of CD11b^hi^ F4/80^+^ macrophages (upper). Cumulative data of CD11b^hi^ F4/80^+^ macrophages (lower); *n* = 3 per group. Data represent mean ± SEM. *n*.s., not significant, Student’s *t*-test. (**D**) Confocal microscopy image of colon section from untreated (CT) and 1.5% DSS-treated WT mouse; sections were stained with antibodies against Ninj1 (red), F4/80 (green), and nuclei (DAPI, blue). Scale bars, 50 μm. Boxed area is magnified on right. CT, control.

Among the immune cells, myeloid cells have been reported to predominantly express Ninj1 during experimental stimulation [3,33]. A previous report indicates that Ninj1 is mainly expressed in myeloid cells isolated from human peripheral blood leukocytes, whereas B and T lymphocytes express relatively low levels of Ninj1 [3]. To define the immune compartment expressing Ninj1 and modulating colonic inflammation, we evaluated Ninj1 expression in bone marrow-derived macrophages (BMDMs), T cells, and B cells. In the naïve state, Ninj1 is highly expressed in BMDMs, as compared to the lymphoid populations (including CD4^+^ and CD8^+^ T cells, and B cells) (Figure 3A, left). During inflammation, leukocytes are differentiated and activated to function appropriately. In the activated state, macrophages reveal highest expression of Ninj1, compared to other lymphocytes (Figure 3A, right). We therefore investigated alteration of Ninj1 expression in macrophages during development of experimental colitis. We observed that mice treated with DSS showed increased Ninj1 expression in peritoneal macrophages (Figure 3B). During the development of intestinal inflammation, macrophage infiltration is vital for pathogenesis. Interestingly, we did not observe decreased prevalence of CD11b^hi^ F4/80^+^ macrophages in the colon tissues of Ninj1-deficient mice (Figure 3C). DSS treatment resulted in marked infiltration of macrophages into the colonic tissues of both Ninj1-deficient and WT mice (Figure 3C). Using immunofluorescent staining, we further confirmed that there were no changes in the number of F4/80^+^ macrophages in the colon tissues from DSS-treated WT and Ninj1 KO mice (Figure S1). To investigate Ninj1 expression in the colon during colitis, the tissues were subjected to immunohistochemical analysis. We observed Ninj1 staining in a subpopulation of cells that are also positive for the macrophage marker F4/80 (Figure 3D). These findings indicate that Ninj1 expressed on macrophages does not modulate the migration capacity of macrophages. Our results suggest that macrophages are responsible for expressing Ninj1 in colon tissues, which exhibit increased Ninj1 during colitis.

### 2.4. Ninj1 in Macrophages Enhances Production of Cytokines Modulating Colon Inflammation

**Figure 4:**
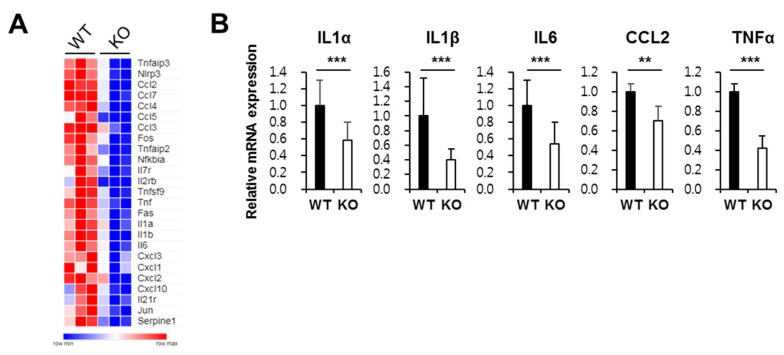
Differences in mRNA expression profiles of macrophages from WT and Ninj1 KO mice (**A**) Heat map of inflammation-related gene expression in peritoneal macrophages from WT and Ninj1 KO mice based on microarray analysis; *n* = 3 per group. (**B**) qRT-PCR was performed to confirm the genes of interest identified by microarray analysis. Data are presented as mean ± SEM. ** *p* < 0.01, *** *p* < 0.005, Student’s *t*-test.

**Figure 5:**
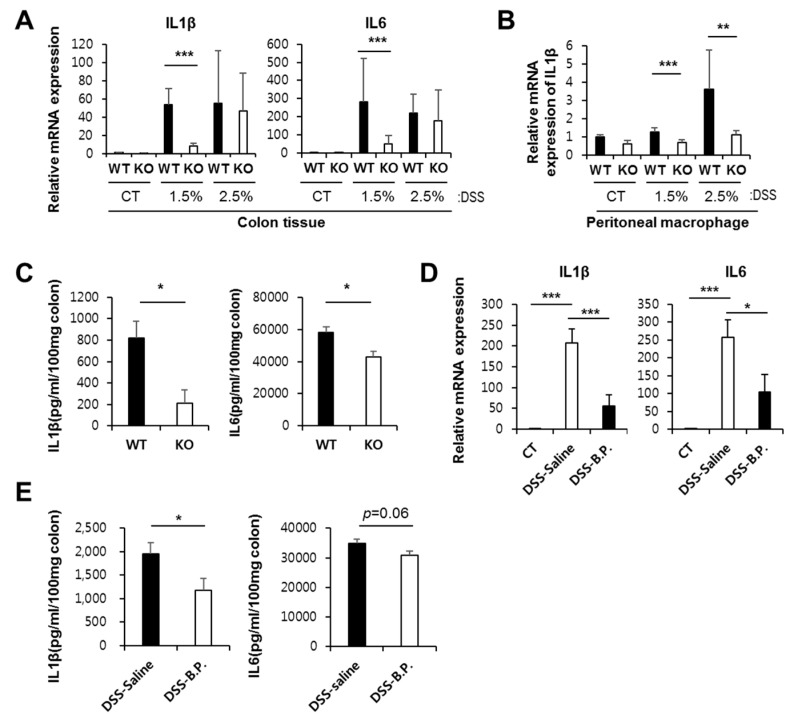
Loss of Ninj1 from macrophages reduces the production of cytokines. (**A**) Relative mRNA expressions of IL1β and IL6 were analyzed by performing qRT-PCR using colon tissues from untreated and DSS-treated WT and Ninj1 KO mice; *n* = 3 per group. The expression values are normalized to GAPDH, and the plots are presented as mean ± SEM. *** *p* < 0.005, Student’s *t*-test. Similar results were observed in three independent experiments. (**B**) Relative mRNA expression of IL1β was analyzed by performing qRT-PCR using peritoneal macrophages from untreated and DSS-treated WT and Ninj1 KO mice; *n* = 3 per group. Data are presented as mean ± SEM. ** *p* < 0.01, *** *p* < 0.005, Student’s *t*-test. (**C**) WT and Ninj1 KO mice were treated with 1.5% DSS for 8 days. Mice were sacrificed on day 8 after initiating DSS treatment. Colonic tissue explants were harvested, and secreted IL1β and IL6 levels were assessed by ELISA; *n* = 4 per group. Data are presented as mean ± SEM. * *p* < 0.05, Student’s *t*-test. Similar results were observed in three independent experiments. (**D**) WT mice were intravenously injected with 0.9% NaCl (saline) as control, or Ninj1 blocking peptide (B.P.), and treated with 1.5% DSS for 8 days. Mice were sacrificed on day 8 after initiating DSS treatment. IL1β and IL6 mRNA expressions in colon tissue were analyzed by qRT-PCR; *n* = 4, untreated; *n* = 5, DSS + saline; *n* = 6, DSS + Ninj1 blocking peptide. Data are presented as mean ± SEM. *** *p* < 0.005, Student’s *t*-test. (**E**) Colonic tissue explants were harvested, and levels of secreted IL1β and IL6 were assessed by ELISA; *n* = 5, DSS + saline; *n* = 6, DSS + Ninj1 blocking peptide. Data are presented as mean ± SEM. * *p* < 0.05, Student’s *t*-test. CT, control.

To elucidate the role of Ninj1 in macrophages and in inflammatory stimuli, we compared gene expressions in peritoneal macrophages from WT and Ninj1 KO mice. Several studies suggest that peritoneal macrophages are implicated in the development of colitis, even though these cells are not present at the inflammatory site [34,35]. Here, we determined that pro-inflammatory cytokines and chemokines such as IL1β, IL6, and CCL2 are upregulated in peritoneal macrophages from WT mice compared to the levels in peritoneal macrophages from Ninj1 KO mice (Figure 4A). Using qRT-PCR, we validated differences in the gene expression of pro-inflammatory cytokines in peritoneal macrophages (Figure 4B). We also found that Ninj1 KO Raw 264.7 cells stimulated with LPS show decreased mRNA expression of IL1β, whereas LPS-treated WT cells exhibit significantly increased IL1β levels (Figure S2). Moreover, overexpression of Ninj1 in THP-1 cells increased mRNA expressions of IL1β and IL6 (Figure S3). Interestingly, we could not detect any changes in the mRNA expression of anti-inflammatory cytokines in IL4-treated BMDM from WT and Ninj1 KO mice (Figure S4). Furthermore, examining the gene expression of a cytokine in colons of DSS-treated mice revealed a decrease in mRNA expressions of IL1β and IL6 in colons of DSS-treated Ninj1 KO mice compared to DSS-treated WT mice (Figure 5A). In peritoneal macrophages from DSS-treated Ninj1KO mice, diminished IL1β expression was obtained, as compared to peritoneal macrophages from DSS-treated WT mice (Figure 5B). To confirm the above results, colon explant culture was performed, and the amounts of IL1β and IL6 was determined by ELISA. The amounts of IL1β and IL6 expressed in the colon explant culture media from WT mice was significantly higher than Ninj1 KO explants (Figure 5C). Furthermore, we examined whether inhibition of Ninj1 affects the intestinal inflammation. WT mice were treated with the blocking peptide, Ninj1_26–37_, to inhibit Ninj1. The mRNA expressions of IL1β and IL6 were dramatically reduced by inhibiting Ninj1 with the blocking peptide during the development of colitis (Figure 5D). The amounts of IL1β in colon explant cultures from Ninj1_26–37_- treated mice with DSS was also lower than that obtained from mice treated with DSS alone (Figure 5E left). Although the difference between the two groups was not significant, there was a trend toward decreased IL6 expression in Ninj1_26–37-_ treated mice with DSS compared to mice treated with DSS alone (Figure 5E right). These results suggest that Ninj1 inhibition decreases the intestinal inflammatory response. Collectively, our results indicate that Ninj1 expression contributes to the activation of macrophages, thereby eliciting a pro-inflammatory response during colitis.

### 2.5. Ninj1 Deficiency in Myeloid Cells Decreases Susceptibility to Experimental Colitis

**Figure 6:**
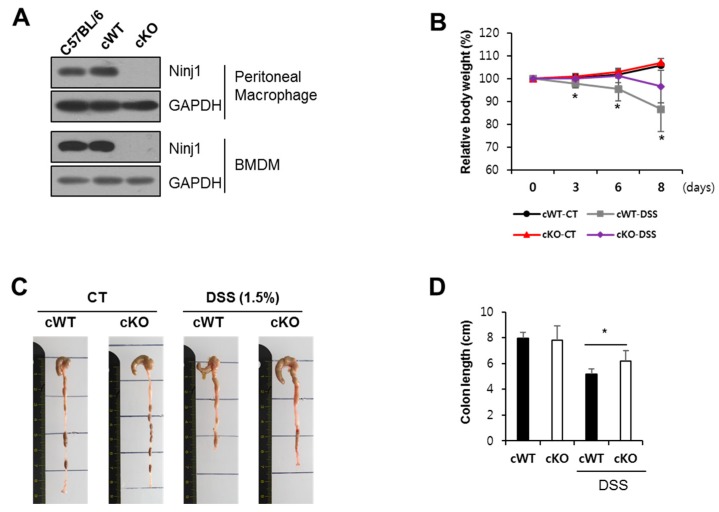
Ninj1 deficiency on macrophages reduces colonic inflammation during DSS treatment. (**A**) Peritoneal macrophages and bone marrow-derived macrophages were isolated from cWT (conditional wild-type: Ninj1^fl/fl^) and Ninj1 cKO (conditional KO: Ninj1^fl/fl^; Lyz-Cre+); Ninj1 was detected by immunoblotting, using GAPDH as the internal control. (**B**) cWT and Ninj1 cKO were administered 1.5% DSS for 8 days; *n* = 5, normal group; *n* = 8, DSS group. Bodyweight loss is presented as a percentage of initial weight (mean ± SD). Student’s *t*-test comparing 1.5% DSS treated cWT mice and 1.5% DSS treated Ninj1 cKO mice, * *p* < 0.05. Similar results were observed in four independent experiments. (**C**) Representative images of colons from cWT and cKO mice. (**D**) Colon lengths were quantified. Data are presented as mean ± SD. * *p* < 0.05, Student’s *t*-test.

**Figure 7:**
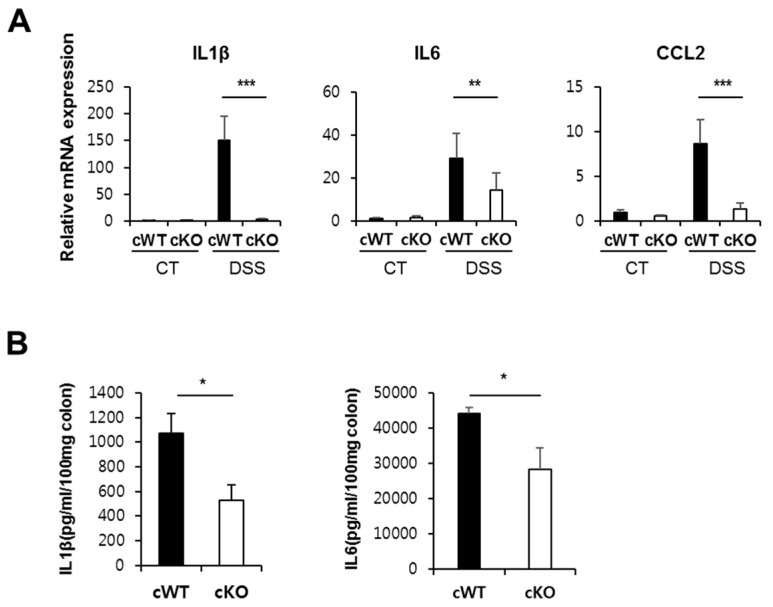
Ninj1 deficiency on macrophages diminishes production of cytokines during DSS treatment. (**A**) Relative mRNA expressions of IL1β, IL6, and CCL2 extracted from colons of cWT and cKO mice. Data are presented as mean ± SEM. ** *p* < 0.01, *** *p* < 0.005, Student’s *t*-test. (**B**) Colonic tissue explants were harvested, and IL1β and IL6 secretions were assessed by ELISA; *n* = 4 per group. Similar results were observed in two independent experiments. Data are presented as mean ± SEM. * *p* < 0.05, Student’s *t*-test. CT, control.

To confirm that Ninj1 in macrophages has an important function in colon inflammation, we generated mice with a myeloid cell-specific Ninj1 deficiency and studied the pathogenesis of colitis. Ninj1 cWT (conditional wild-type: Ninj1^fl/fl^) mice were crossed with Lyz-Cre mice to generate Ninj1 conditional KO mice (Ninj1^fl/fl^; Lyz-Cre+ mice, designated as Ninj1 cKO). Western blot analysis validated Ninj1 expression in peritoneal macrophages and BMDM of the Ninj1 cKO mice (Figure 6A). We also examined intestinal inflammation in cWT and Ninj1 cKO mice; the cWT mice exhibit significant bodyweight loss, compared with Ninj1 cKO mice (Figure 6B). The cWT mice also show marked colon shortening (Figure 6C,D). Relative mRNA expressions of IL1β, IL6, and CCL2 were significantly lower in the colon of DSS-treated Ninj1 cKO mice than cWT mice (Figure 7A). The amounts of IL1β and IL6 expressed in the colon explant culture media from cWT mice were higher than that obtained from Ninj1 cKO mice, which is consistent with results of mRNA expression analysis (Figure 7B). These results indicate that absence of Ninj1 in myeloid cells is sufficient to alleviate experimental colitis.

### 2.6. Ninj1 Modulates PKCδ/θ Activation

**Figure 8:**
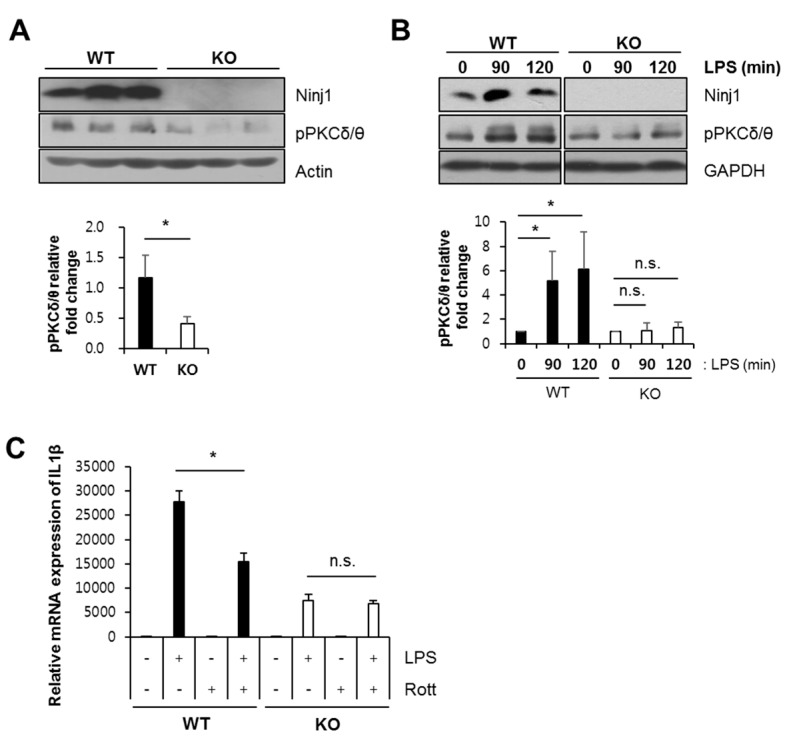
The PKCδ/θ activation is regulated by Ninj1 in macrophages. (**A**) Immunoblot analysis of Ninj1, pPKC δ/θ, and Actin performed on colonic tissues extracted from 1.5% DSS-treated WT and Ninj1 KO mice; *n* = 3 per group. Densitometry represents relative protein levels of pPKC δ/θ. Values presented are mean ± SD. * *p* < 0.05, Student’s *t*-test. (**B**) WT and Ninj1 KO Raw264.7 cells were treated with 0.5 μg/mL of LPS for 2 h. Western blot analysis was performed to detect the expression of Ninj1, pPKCδ/ θ, and GAPDH. Densitometry represents relative protein levels of pPKC δ/θ. n.s., not significant. Values presented are mean ± SD. * *p* < 0.05, Student’s *t*-test. Similar results were observed in three independent experiments. (**C**) WT and Ninj1 KO Raw264.7 cells were treated with 5 μg/mL of rott (rottlerin) and LPS (0.5 μg/mL) for 6 h. qRT-PCR was performed to detect mRNA expressions of IL1β. Data are presented as mean ± SEM. * *p* < 0.05, Student’s *t*-test. Similar results were observed in three independent experiments.

Western blot analysis was performed to identify the molecular pathway by which Ninj1 modulates PKCδ/θ activation. PKCδ/θ in colons of DSS-treated WT mice show increased activation levels, compared to DSS-treated KO mice (Figure 8A). To verify this result, we administered LPS to WT and Ninj1 KO Raw 264.7 to mimic macrophage activation during development of colitis. In WT cells, LPS stimulation significantly increased the activation of PKCδ/θ (Figure 8B). However, LPS-treated Ninj1 KO cells showed no difference in the activation of PKCδ/θ compared to WT cells (Figure 8B). Moreover, LPS-administered WT and Ninj1 KO Raw264.7 cells were treated simultaneously with rottlerin (rott), a PKC δ/θ inhibitor, to examine the IL1β mRNA expression levels. As expected, LPS-treated WT Raw264.7 cells show markedly increased mRNA expression of IL1β compared to LPS-treated Ninj1 KO Raw264.7 cells (Figure 8C). Treatment with rott decreases the mRNA expression of IL1β in LPS-treated WT Raw264.7 cells, as compared to LPS treatment alone (Figure 8C). However, in Ninj1 KO cells, almost no change was observed with combined treatment of rott and LPS, as compared to cells treated with LPS alone (Figure 8C). Taken together, these results indicate that Ninj1 regulates PKCδ/θ activation, leading to the production of cytokines.

## 3. Discussion

The results of this study illustrate that Ninj1 in macrophages is a regulatory factor involved in the development of colitis. Using myeloid cell-specific Ninj1-deficient mice as well as conventional Ninj1 KO mice, we show that the loss of Ninj1 attenuates intestinal inflammation. These findings clearly demonstrate that a deficiency of Ninj1 in macrophages decreases intestinal inflammation, resulting in reduced pathogenesis.

In recent years, several papers have reported that Ninj1 expression is induced under inflammatory conditions [2,3,4,36]. In accordance with these previous results, we identified that protein expression of Ninj1 is induced in inflamed colon tissues of WT mice. We presumed that the increased expression of Ninj1 in inflamed colon homogenates is the result of induction of its expression on macrophages. Although macrophage infiltration is shown to be a critical step in the development of colitis, there were no changes in the numbers of macrophages extracted from colons of DSS-treated WT and Ninj1 KO mice. Ninj1-deficient and Ninj1-expressing macrophages have similar capacities to migrate to the site of a lesion, thereby suggesting that the difference in colitis incidences seen in WT and Ninj1 KO mice are not determined by differences in macrophage infiltration, but rather by activation of macrophages. These results conflict a previous report dealing with the role of Ninj1 in colon cancer. During colon cancer development, overexpression of Ninj1 suppresses the migration of macrophages, resulting in the alleviation of cancer development [37]. Macrophages in tumor tissue directly facilitate the enhancement of growth and mobility of tumor cells. As a tumor progresses, cancer cells train macrophages to adopt new characteristics, allowing them to contribute to migration, angiogenesis, intravasation, and extravasation [38,39]. Thus, tumor-associated macrophages (TAM) function in differently than macrophages of normal tissue. Moreover, we analyzed previously published mRNA profiles and determined that Ninj1 expression is not induced in colon cancer tissues of mice, as compared to mice with normal colons (GSE31106) [40]. In this paper, we present the different roles of Ninj1 in macrophages under intestinal inflammatory conditions as compared with TAM during colon cancer development.

Similar to the previous paper that analyzed the role of Ninj1 in colon cancer, several reports have demonstrated that Ninj1 mediates migration of cells in inflammatory conditions [2,3,4,41,42,43]. Thus, it was surprising that during colitis development, Ninj1 did not alter the migration capacity of macrophages, which is seen only in lung fibrosis. Upon bleomycin treatment, Ninj1 KO mice ameliorated lung fibrosis; however, the number of infiltrating macrophages from both KO and WT mice were not different [5]. During development of colitis and lung fibrosis, the Ninj1 pathway controlled activation of the macrophages, resulting in the triggering an inflammatory response.

The interaction of LPS and macrophage is important in the pathogenesis of DSS-induced colitis model [44]. LPS-activated macrophages in colon tissues trigger the onset of mucosal edema and diarrhea [45]. LPS is a well-known activator of TLR4 on macrophages, and for full activation of inflammatory signals, PKCδ/θ is considered an important factor in the TLR4-mediated pathway [26,27]. In the current study, the combined treatment with PKCδ/θ inhibitor and LPS resulted in diminished cytokine secretion, as compared to LPS-alone treatment group (Figure 8C). Therefore, we assume that Ninj1 regulates TLR4-PKCδ/θ activation under inflammatory conditions.

We also present evidence that our results from the murine colitis model are relevant to human IBD patients. By analyzing published data, we detected a correlation between Ninj1 expression and IBD (Figure 1A). Furthermore, the transcriptional profile in peripheral blood mononuclear cells (PBMC) revealed that mRNA expression of Ninj1 is increased in PBMC of IBD patients, as compared to levels obtained in PBMC of healthy subjects (Figure S5) [46]. Macrophage is one of the cell types present in PBMCs, and myeloid cells in human PBMC are known to present the highest levels of Ninj1 expression among the leukocytes [3]. Thus, we deduce that there is a positive correlation between Ninj1 and IBD.

Over-active immune response is one of the main etiologies that cause IBD [47]. Immunosuppressive agents have been considered as the therapeutic focus. Infliximab, adalimumab, and natalizymab are beneficial in the treatment of CD and UC [48,49,50]. According to a previous report, treatment of Ninj1 blocking peptide diminishes systemic inflammation in septic mice [4]. In the current study, we observed that the attenuated gut inflammation in mice treated with Ninj1 blocking peptide compared to untreated mice during DSS treatment, which exhibits its potential as an immunosuppressive therapy.

Epithelial barrier destruction is associated with IBD. Tight junction proteins are located in the apical region of the intestinal epithelium, and form selectively permeable barriers. Increased permeability and loss of tight junction-related proteins are prerequisites for the development of colitis [51]. When mimicking the intestinal barrier in vitro, the Caco2 cell line is the most popular model because of its ability to spontaneously differentiate into an enterocyte-like phenotype. We evaluated Ninj1 expression in Caco2 cells and detected a relatively low expression level (Figure S6). This result was parallel to our immunofluorescence data which reveals that Ninj1 expression is mainly detected in non-epithelial cells (Figure 1D). We subsequently overexpressed Ninj1 in Caco2 cells with Ninj1 expressing adenovirus and subjected the cells to immunofluorescence staining with the anti-Zo-1 antibody. Regular distribution of Zo-1 protein in a Caco2 monolayer was unaffected by the overexpression of Ninj1 (Figure S7). DSS was applied to Caco2 monolayers to mimic intestinal barrier dysfunction; this treatment disrupted the Zo-1 distribution in the Caco2 monolayer, with no significant changes in Zo-1 distribution upon Ad-Ninj1 treatment (Figure S7). Overexpression of Ninj1 in intestinal epithelial cells did not alter the intestinal barrier function. This result supports our hypothesis that non-epithelial cells are involved in the different phenotypes of the DSS-treated WT and Ninj1 KO mice.

In conclusion, our results demonstrate that Ninj1 in macrophages contributes to intestinal inflammation in experimental colitis. Therefore, we propose that targeting Ninj1 may be a promising therapeutic approach to decreasing the pathogenesis of colitis.

## 4. Materials and Methods

### 4.1. Mice and Experimental Colitis

Ninj1 KO C57BL/6 mice were generated as previously described [5]. To generate myeloid-specific Ninj1-deficient mice, Ninj1^fl/fl^ mice were bred with heterozygous Lyz2-cre+/− mice. Male and female (9 to 11-weeks-old) mice were randomly assigned to experimental groups. Colitis was induced in mice by administering 1.5% or 2.5% (w/v) DSS (36–50 kDa) (MP Biomedicals, OH, USA) in drinking water for 8 days. Mice were weighed every 2~3 days after initiation of DSS treatment, and subsequently sacrificed on day 8 after initiation of treatment. Animals were euthanized by carbon dioxide inhalation, and the colon tissues were harvested to determine the colitis incidence. For blocking peptide treatment, Ninj126-37 peptide (1 mg/kg) was diluted in 0.9% NaCl and administered intravenously immediately before initiation of DSS treatment. All animal experiments were conducted in accordance with the protocols approved by the Institutional Animal Care and Usage Committee at Gachon University in Korea (GIACUC-R2016005, 2016.08.20; GIACUC-R2017014, 2017.05.24; GIACUC-R2018006, 2018.05.21).

### 4.2. Cell Lines

Raw264.7 cells were cultured in DMEM supplemented with 10% FBS (Welgene, Daegu, Korea). Using CRISPR Cas9, Ninj1-deficient cells were generated as previously described [5]. In the current study, Ninj1-expressing cells are denoted as WT cells, while Cas9-treated Ninj1 KO cells are referred to as Ninj1 KO cells. The WT and Ninj1 KO Raw264.7 cells were treated with LPS (Sigma-Aldrich, St Louis, MO, USA) or rottlerin (rott; Santa Cruz Biotechnology, CA, USA). Human monocytic cell line (THP-1) was purchased from ATCC and cultured in RPMI 1640 supplemented with 10% FBS. THP-1 cells were transfected with adenovirus expressing human Ninj1 (Ad-Ninj1) or empty vector (Ad-EV), as previously described [42]. Human colon carcinoma cell line (Caco2) was purchased from the Korea Cell Line Bank (Seoul, Korea) and cultured in DMEM supplemented with 10% FBS.

### 4.3. Immunoblotting

As previously described, Western blot analysis was performed against anti-Ninj1 (Abclon, Seoul, Korea), anti-pPKCδ/θ (Cell Signaling Technology, MA, USA), anti-actin, and anti-GAPDH (Millipore, Schwalbach, Germany) [52]. Briefly, cell lysates were prepared with modified RIPA buffer containing protease and phosphatase inhibitors, and equivalent amounts of protein were resolved in sodium dodecyl sulfate-polyacrylamide gels. The proteins were transferred to polyvinylidene fluoride membrane. After the blot was incubated with primary and HRP-conjugated secondary antibodies, the resultant protein-antibody complexes were analyzed using chemiluminescence Western blotting detection reagents (GE Healthcare, Chalfont St. Giles, UK).

### 4.4. Assessment of Inflammation in Colon

Entire colons were fixed overnight in 10% neutral buffered formalin, and subsequently embedded in paraffin. Histology was evaluated after longitudinal sections of colons (swiss-roll) were stained with H&E. Histopathological evaluation assigned scores ranging from 0 to 3 for submucosal edema (0–3), surface epithelial erosion (0–3), and crypt damage (0–3), for a combined total score of 9; submucosal edema: 0 = no significant edema, 1 = rare areas of submucosal edema, 2 = occasional areas of mild submucosal edema, 3 = frequent areas of marked submucosal edema; surface epithelial erosion: 0 = none, 1 = rare small breaches in epithelium, 2 = frequent small breaches in epithelium, 3 = extensive areas lacking surface epithelium; crypt damage: 0 = none, 1 = some crypt damage, 2 = large areas without crypts, 3 = no crypts.

### 4.5. Immunofluorescence

For immunofluorescence assessment, colon sections were deparaffinized, hydrated with distilled water, and incubated in a blocking solution (Life Technologies, MD, USA) for 1 h at room temperature (RT). The primary antibody against Ninj1 (Abclon) was diluted 1:300 and applied to the section-containing slides at RT for 1 h. The bound primary antibodies were detected using 1:300 AlexaFluore568. To assess co-localization of Ninj1 and F4/80, 1:500 rat F4/80-FITC (eBioscience, CA, USA), was incubated with the slides, overnight at 4 °C, followed by staining with Ninj1 antibodies. The nucleus was stained, and the slides were mounted with Vectashield mounting medium (Vector Laboratories, CA, USA).

### 4.6. Immunohistochemistry

Formalin-fixed paraffin-embedded tissue sections of colons from DSS-treated WT and Ninj1 KO mice were deparaffinized and rehydrated prior to ki-67 staining (Abcam, MA, USA). Slides were incubated with antibody against ki-67 for 1 h at RT, followed by incubation with the secondary antibodies for 1 h at RT. Finally, the slides were incubated with DAB substrate (DAKO, CA, USA) and counterstained with hematoxylin. For Alcian blue staining, the slides were stained in Alcian blue solution for 30 min and subsequently washed with distilled water. Counterstaining was conducted with nuclear fast red solution for 5 min, after which the slides were washed and mounted.

### 4.7. Purification and Activation of Lymphocytes

A single cell suspension was isolated from the spleen of C57BL/6 mice. CD4+ and CD8+ T cells were purified using CD4 and CD8 microbeads, in accordance with the manufacturer’s instructions (Miltenyi Biotech, Gladbach, Germany). B cells were purified using CD43 microbeads with MACS kit. CD4+ and CD8+ T cells were activated with plate-bound anti-CD3 (5 μg/mL) and soluble anti-CD28 (5 μg/mL) supplemented with IL-2, and cultured for 3 days. B cells were stimulated with BAFF (50 ng/mL) and LPS (10 μg/mL) for 3 days.

### 4.8. RNA Isolation and Reverse Transcription–Polymerase Chain Reaction (RT-PCR)/Real-Time Quantitative PCR (qRT-PCR) Analysis

Total RNA was isolated from colon tissues and cells using the TRIzol reagent (Invitrogen, CA, USA). Using a synthesis kit, the extracted RNA was used as a template for reverse transcription to synthesize cDNA according to the manufacturer’s instructions (Takara, Kyoto, Japan). The RT-PCR products were analyzed on 1.5% agarose gel in Tris-acetate/ethylenediaminetetraacetic acid buffer. qRT-PCR was performed with the Stratagene Mx3000P QPCR System (Agilent Technologies, La Jolla, CA, USA). The primers for PCR are indicated in Supplementary Table S1.

### 4.9. Cell Isolation from Colons

Colons were dissected from mice and washed with cold phosphate-buffered saline (PBS). Colons were then cut into small pieces, inverted, and incubated at 37 °C in RPMI supplemented with 0.015% DTT and 1 μM EDTA for 30 min with gentle shaking. The remaining tissue was incubated at 37 °C in RPMI supplemented with 1.5 mg/mL collagenase and 0.5 mg/mL dispase, with gentle shaking. The supernatant containing the lamina propria cells was passed through a 70 μm strainer and stained for use in flow cytometric analysis.

### 4.10. Flow Cytometric Analysis

Lamina propria cells were stained with fluorochrome-conjugated antibodies against CD45.2, CD11b and F4/80 (BD Bioscience, CA, USA). A FACS Canto II instrument (BD Biosciences) was used to acquire the results, which were analyzed by FlowJo V10 software (Tree Star Inc, OR, USA).

### 4.11. Peritoneal Macrophage Isolation

Peritoneal macrophages were harvested from the peritoneal cavity using a syringe. The cells were incubated at 37 °C for 40 min in cell culture plates. After non-adherent cells were washed, the adherent cells were collected and prepared for further experiments.

### 4.12. Bone Marrow-Derived Macrophage (BMDM) Isolation and Culture

Cells were flushed from the femur of mice and incubated in RPMI1640 medium (Welgene, Daegu, Korea) supplemented with 10% FBS and 30 ng/mL of mouse colony-stimulating factor (Miltenyi Biotec, Gladbach, Germany). After 5 days, adherent cells were harvested and cultured in new plates for use in further experiments. To stimulate BMDM, 0.5 μg/mL of LPS was applied for 2 h, or 10 ng/mL of IL4 (Peprotech, London, UK) was applied for 12 h.

### 4.13. Microarray Analysis

RNA was isolated from peritoneal macrophages extracted from WT and Ninj1 KO mice. RNA from each sample was synthesized to make cDNA by using the GeneChip Whole Transcript amplification kit, according to the manufacturer’s protocol. The cDNA was hybridized with the Affymetrix Mouse Gene ST 2.0 arrays and analyzed by Macrogen (Seoul, Korea). Data were normalized by applying the multi-average (RMA) method, and statistical significance was calculated by applying the LPE test.

### 4.14. Colon Explant and ELISA

Approximately 1 cm of colon tissue was excised and washed several times with PBS supplemented with penicillin and streptomycin. The tissue was then incubated in RPMI 1640 media in 24-well plates at 37 °C for 24 h. The supernatant was collected and centrifuged to remove debris. Using a DuoSet mouse IL1β and IL6 ELISA, the supernatants were analyzed for IL1β and IL6 content according to the manufacturer’s instructions (R&D Systems).

### 4.15. Analysis of Tight Junction Protein Complex in Vitro

Caco2 cells were seeded on sterile coverslips at 90~100% confluency, and allowed to differentiate for an additional 10 days. The differentiated cell monolayers were transfected with adenovirus expressing human Ninj1 (Ad-Ninj1) or empty vector (Ad-EV), with or without 5% DSS, as previously described [42]. The cells were fixed and incubated with ZO-1 antibody, as the protocol published in a previous report [53].

### 4.16. Statistical Analysis

Results are presented as the mean ± SD or mean ± SEM. When comparing two groups, statistical significance was determined by applying the two-tailed Student’s *t*-test. Ninj1 gene expression data were retrieved from the microarray data matrices uploaded on GEO by the original authors (GSE1710, GSE22307, and GSE3365). R scripting was applied to obtain the expression values of Ninj1 from data matrices. Welch’s T test was utilized to compare Ninj1 gene expressions in different specimens. Testing was performed using the SPSS20 23.0 software (SPSS, IL, USA). Significance of difference *p*-values are represented in text and figures as * *p* < 0.05, ** *p* < 0.01, or *** *p* < 0.005.

## Supplementary Materials

Supplementary materials can be found at https://www.mdpi.com/1422-0067/21/2/614/s1.
