# Supplementary material for: Detrimental Role of Nerve Injury-Induced Protein 1 in Myeloid Cells under Intestinal Inflammatory Conditions
Source: Int J Mol Sci. 2020 Jan 17;21(2):614. doi: 10.3390/ijms21020614 (PMC7013940; doi:10.3390/ijms21020614)
Supplement: Supplementary file 1 [file ijms-21-00614-s001.pdf]

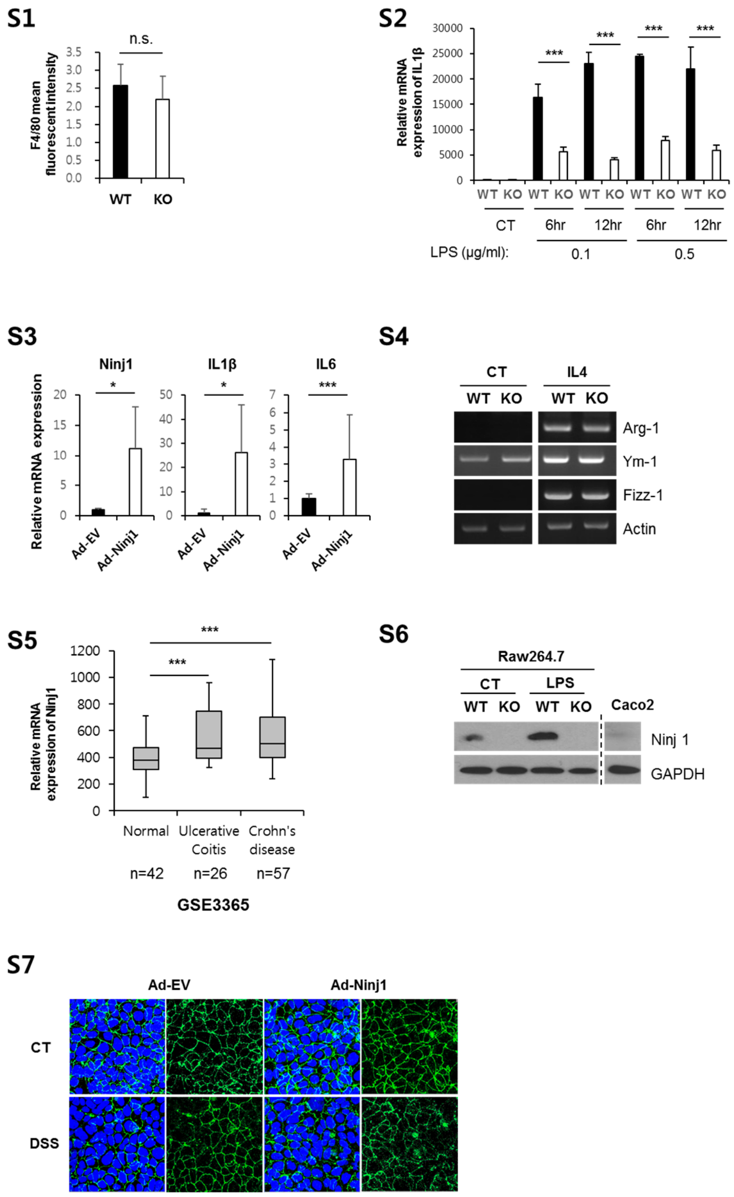

Supplementary Figure S1. Immunofluorescence staining of F4/80 in colon tissue sections from mice. The quantification of F4/80 immunofluorescence is presented as the mean fluorescence intensity; n=3 per group. Values are mean ± SD. Student's t-test. Not significant (n.s.).

Supplementary Figure S2. Ninj1 in Raw264.7 cells regulate the inflammatory response. WT and Ninj1 KO Raw264.7 cells were treated with 0.1 or 0.5 μg/mL of LPS for the indicated duration. The mRNA expression of IL1β was detected and normalized by GAPDH. Data are presented as mean ± SEM. ***$p < 0.005$, Student's t-test. CT, control.

Supplementary Figure S3. Overexpression of Ninj1 increases cytokine production. THP-1 cells were infected with Ad-EV (adenovirus-empty vector) or Ad-Ninj1. The mRNA expressions of Ninj1, IL1β, and IL6 were detected by qRT-PCR and normalized by GAPDH. Data are presented as mean ± SEM. *$p < 0.05$, ***$p < 0.005$, Student's t-test.

Supplementary Figure S4. Anti-inflammatory cytokines were detected by RT-PCR. Bone marrow-derived macrophages (BMDM) were extracted from WT and Ninj1 KO mice incubated with 10 ng/mL of IL4 for 12 h. CT, control.

Supplementary Figure S5. Analysis of Ninj1 expression in peripheral blood mononuclear cells from UC and CD patients. The gene expression data (GSE3365) were obtained from GEO database. ***$p < 0.01$, Welch's T test.

Supplementary Figure S6. Ninj1 expression in Raw 264.7 and Caco2 cells was detected by Western blotting. CT, control.

Supplementary Figure S7. The effect of Ninj1 on the structure of tight junction complexes *in vitro*. Caco2 cell monolayers were infected with Ad-EV (adenovirus-empty vector) or Ad-Ninj1, and subsequently incubated with 5% DSS. Cell monolayers were stained for ZO-1 and images were collected by confocal microscopy. ZO-1 (green), nuclei (DAPI, blue), and CT, control.

Supplementary Table S1. PCR primer list.

**Genes**	**Species**	**Direction**	**Primers (5' ->3')**
Ninj1	Mouse	Forward	GAGTATGAGCTCAACGGCGA
		Reverse	TGACCAGGAAGATGAGCAGC
IL1β	Mouse	Forward	GCCTTGGGCCTCAAAGGAAAGAATC
		Reverse	GGAAGACACAGATTCCATGGTGAAG
CCL2	Mouse	Forward	TCCCAATGAGTAGGCTGGAGAGC
		Reverse	CAGAAGTGCTTGAGGTGGTTGTG
IL1α	Mouse	Forward	GATGACCTGCAGTCCATAACC
		Reverse	CTGGCAACTCCTTCAGCAAC
IL6	Mouse	Forward	GAGGATACCACTCCCAACAGACC
		Reverse	AAGTGCATCATCGTTGTTCATACA
TNFα	Mouse	Forward	ATAGCTCCCAGAAAAGCAAGC
		Reverse	CACCCCGAAGTTCAGTAGACA
Arg-1	Mouse	Forward	CAGAAGAATGGAAGAGTCAG
		Reverse	CAGATATGCAGGGAGTCACC
Ym-1	Mouse	Forward	AGAAGGGAGTTTCAAACCTGG
		Reverse	TGTTTGTCCTTAGGAGGGC
Fizz-1	Mouse	Forward	TCCAGCTGATGGTCCCAGTGAATA
		Reverse	GGCAGTGGTCCAGTCAAC
Actin	Mouse	Forward	TGGAATCCTGTGGCATCCATGAAAC
		Reverse	TAAAACGCAGCTCAGTAACAGTCCG
GAPDH	Mouse	Forward	AAGGGCATCTTGGGCTACACT
		Reverse	TACTCCTTGGAGGCCATGTAGG
Ninj1	Human	Forward	CAAGCTGGACTTCCTCAACAA
		Reverse	CATGTCCATCAAGGGCTTCT
IL1β	Human	Forward	AGCTGTACCCAGAGAGTCC
		Reverse	ACCAAATGTGGCCGTGGTTT
IL6	Human	Forward	AGACAGCCACTCACCTCTTCA
		Reverse	CACCAGGCAAGTCTCCTCATT
